# Osteogenesis imperfect

**DOI:** 10.11604/pamj.2021.40.98.31815

**Published:** 2021-10-13

**Authors:** Surya Besant Natarajan, Krishna Prasanth Baalann

**Affiliations:** 1 Department of Community Medicine, Sree Balaji Medical College and Hospital, Bharath Institute of Higher Education and Research, Chennai, India

**Keywords:** Osteogenesis imperfecta, brittle bone disease, genetic disease

## Image in medicine

Osteogenesis imperfecta is a rare hereditary connective tissue disorder that affects the bones fragility. It causes the bones to break easily. The defective gene affects the body mechanism to form collagen, which strengthens the bone. A 20-year-old male patient came with complaints of pain in the left leg since morning. He was a known case of osteogenesis imperfecta and already had a history of fracture 6 months back in the right upper limb. On local examination of left leg swelling present, tenderness present, sensations intact and restricted range of motion. X-ray showed fracture of shaft of left tibial bone. The patient was on plaster of Paris for 6 weeks. The patient was advised for regular physiotherapy and to follow-up with the orthopedic surgeon.

**Figure 1:**
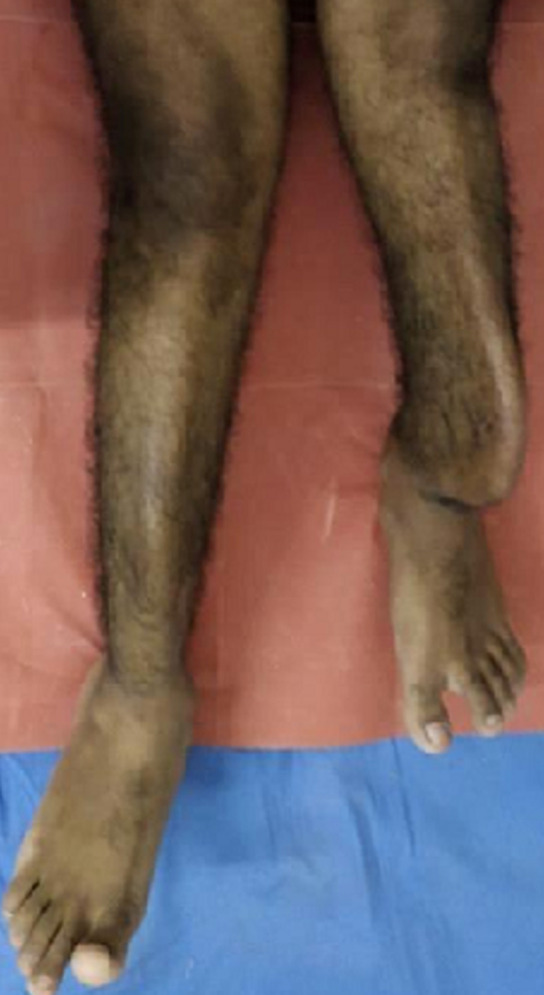
examination findings-protrusion of shaft of left tibial bone

